# Navigating urban congestion: A Comprehensive strategy based on an efficient smart IoT wireless communication for PV powered smart traffic management system

**DOI:** 10.1371/journal.pone.0310002

**Published:** 2024-10-25

**Authors:** Rana Ahmed, Radwa Ahmed Osman, Motaz Amer

**Affiliations:** 1 College of Engineering, Electrical and Control, Arab Academy for Science, Technology and Maritime Transport, Alexandria, Egypt; 2 College of Engineering, Basic and Applied Science, Arab Academy for Science, Technology and Maritime Transport, Alexandria, Egypt; Shanghai Jiaotong University: Shanghai Jiao Tong University, CHINA

## Abstract

Egypt faces extreme traffic congestion in its cities, which results in long travel times, large lines of parked cars, and increased safety hazards. Our study suggests a multi-modal approach that combines critical infrastructure improvements with cutting-edge technologies to address the ubiquitous problem of traffic congestion. Assuring vehicles owners of their timely arrival, cutting down on fuel usage, and improving communication using deep learning approach and optimization algorithm within the potential of IoT enabled 5G framework are the main goals. The traffic management system incorporates detection cameras, Raspberry Pi 3 microcontroller, an Android application, cloud connectivity, and traditional traffic lights that are powered using PV modules and batteries to secure the traffic controllers operation in case of grid outage and assure service continuity. The model examines the difficulties associated with Internet of Things (IoT) communication, highlighting possible interference from device-to-device (D2D) devices and cellular user equipment. This all-encompassing strategy aims to reduce fuel consumption, increase road safety and improve traffic efficiency. The model predicts a significant increase in Egypt’s urban mobility by utilizing the possibilities of IoT and 5G technologies, which would improve Egypt’s towns’ livability and efficiency. The goal of this paper is to modernize Egypt’s traffic management system and bring it into compliance with global guidelines for intelligent transportation networks.

## Introduction

Due to escalations in vehicles rate, the traffic congestion became a worldwide serious problem. Consequently, the road expansion rate is incapable of handling it in most countries. The International Road Federation has reported an increase in the death rate due to traffic accidents and traffic jams at which 20% or more of patients who need grave medical attention deceased on their way to hospitals due to retard in traffic. In addition, traffic congestion has a negative consequence, counting pollution, loss of money due to increased fuel consumption, time loss and unanticipated collisions [1, 2].

To properly manage the amount of traffic on the roads, a smart traffic management system is recommended [3]. In reaction to the vehicle density on a particular roadside, it automatically and dynamically modifies the signal time. By talking with one another and the local network, the traffic lights controlled the flow of traffic, making the system more effective. According to the decentralised method, the optimal dynamic timing based on density estimates and the placement of traffic signals with the best separation distance between them are both effective. When utilising this suggested strategy, the likelihood of traffic congestion caused by the conventional traffic system is minimised. Additionally, simultaneous and ongoing traffic monitoring helps to prevent accidents and traffic infractions.

Embedded sensors, security cameras, and RFIDs (radio frequency identifications) on both the road sides is also intended to control traffic on road networks utilising [4, 5]. The distributed device processes video data at the local server and sensor data at the node level, calculating total density to manage traffic depending on density. It also targets ambulances and fire engines, among other essential vehicles. Additionally, users are able to predict the level of congestion along a route.

Utilizing a (ST-CA) multi-layered architecture, the system in [6] will assist with organizing traffic congestion, particularly at intersections. The wireless sensor network (WSN) is the layer (top layer) that has several sensors in the streets to record the traffic information and the movements of passing cars. The data is sent from the data collection layer (middle layer) to the cloud layer (last layer) after it has been collected. Using a decision-making algorithm, cloud computing is employed to assess the data and traffic density. A global positioning system (GPS) is required for signal transmission and controller communication. By instructing the automobiles to avoid the congested areas instead of driving through them, this method will alleviate the congestion issues.

On the other hand, a smart Road traffic Control management system termed Urban Traffic Control (UTC) is introduced in [7] In order to enhance a current road network and provide a more organised traffic arrangement, UTC is taking into account the entire traffic network rather than just taking intersections into account plus utilizing certain indicators and models. These versions include vehicle priority, lane weight, and traffic jam indicator. A NetLogo stimulator is utilized to verify the idea by comparing the fixed cycle traffic light with the random behaviour that is generated and scattered over 25 different intersections for a time duration of 9 h on both lane flow and no interference movement flow. According to the data, there was a considerable reduction of 34.16% for no interference movement flow and a reduction of 25.98% for the total average waiting time across all vehicles during the simulation period.

The transmission of the data is required to be efficient and reliable in order to increase the road safety and enhance traffic management. Therefore, different models and techniques has been proposed to enhance IoT communication efficiency. The backscatter communication (BC) to connect IoT devices has been used recently in a multicell IoT network where each cell’s source transmits a superimposed signal to associated IoT devices, and backscatter sensor tags (BST) reflect and modulate this signal to transmit a reliable data to IoT devices [8]. Three major 5G service types are outlined by the International Telecommunications Union as helping to improve the communication of IoMT devices: uRLLC (ultra-Reliable Low Latency Communications) for critical, low-latency needs; eMBB (enhanced Mobile Broadband) for high data throughput; and mMTC (massive Machine-type Communications) for numerous devices with quality service. In a cell, these functions are usually provided individually. However, there is a need to efficiently provide different service categories due to the increasing number of connected devices, increased data rates, and energy consumption [9].

Despite all the proposed models in the literature to solve the traffic management issue, still there is a room on how the efficient IoT communication can be implemented in order to obtain the required traffic management. This paper proposed a novel method for traffic management taking into account the required reliable communication between IoT and everything whether it was the driver or the traffic department in order to improve the traffic road conditions. The contribution of this paper is divided into different phases as follows:

- An intelligent, solar-powered traffic control system to assure service continuity. It identifies the traffic density over a particular distance using cameras mounted above the different stop lines. The cameras will detect the road and log data into the Raspberry pi3-microcontroller which will manipulate this data by image processing technique and sends information to the cloud and the android app based on the 5G technology at every time interval. In addition, the microcontroller will automatically modify the traffic light signals according to the vehicle density overreaches a certain distance. At certain identified vehicle density, the traffic signal turns green (vehicles pass); if not, the traffic signal turns red (vehicles await). In consequence, the holding pattern of the traffic light is set and the junction timing modify automatically.
- A new and effective Internet of Things network that dynamically adjusts the transmission power of connected devices to reduce interference. The objective of this innovation is to improve network performance and reliability by reducing interference.
- To increase the reliability of communication between IoT devices and different entities, such as drivers and traffic management systems, an optimization issue is created. In order to maximize network efficiency, this challenge was solved by combining Lagrange optimization techniques with a 1-Deep Convolutional Neural Network (1 D-CNN).
- Our deep learning approach controls interference from other devices and forecasts the ideal transmission power required for Internet of Things devices. The network’s efficiency and dependability are greatly increased by this feature.
- IoT is utilized to keep automobiles and infrastructure (such as traffic signals and road sensors) in communication. Through the provision of fast updates and modifications to traffic signals and management systems, IoT devices enable real-time data interchange and coordination, which improves traffic flow and reduces congestion.

The proposed approach was investigated in challenge to avoid the limitations of the traditional traffic lights that frequently have fixed timing and do not adapt to changing traffic patterns, which causes needless delays and congestion. Furthermore, insufficient utilization of available data from cameras and sensors by conventional systems may lead to insufficient traffic management. In addition, inefficient traffic flow and longer travel times are frequently the result of traditional techniques’ inability to adjust in real time to changing traffic conditions. The remaining paper is further arranged in such a manner that section 2 presents related work. The proposed model is discussed in section 3. Section 4 shows the simulation results. Finally, section 5 covers the conclusion of the paper.

## Related work

A variety of contributions were introduced by the researchers to tackle the problem of traffic management. In [10]., a smart barrier system was employed for traffic control. The fundamental idea behind this intelligent system is the localisation of barricades depending on the environment and the volume of traffic in relation to three different barrier modes: open, close, and partially open. In order to create the smart barrier system for dynamic traffic control, historical data must first be gathered. Adopting various traffic centres that make it easier to collect the number of cars moving at a given time and their speeds allows for the gathering of historical data. The fuzzy logic system in this model decides where to place the barrier and also focuses on determining the best membership limitations to produce better outcomes.

A hybrid intelligent traffic management system and a new smart traffic signal controller was proposed in [11] to avoid the problems (include inadequate time management at road crossings; they are not resistant to certain climatic circumstances, such as rain; and they lack the ability to prioritise emergency vehicles) of the traditional tri-colour signals system. The suggested technique is based on Vehicular Ad-hoc Networks and Internet of Vehicles technologies, which allow vehicles to wirelessly connect with one another and with a dedicated infrastructure. The suggested adaptive algorithm not only decreases the average delay, yet it also improves the quantity of serviced vehicles. Although there isn’t a clear definition for the Internet of Things because it’s a relatively new phenomena, it may be generally characterised using this relation [12, 13].


IoT=physicalentity+controllers,sensors,actuators+internet.

With the development of technology and the proliferation of sensors in nearly every device, huge amounts of data must be captured, processed, saved, and presented in a way that is easy to understand. IoT data that would normally take months to process may be used in only a few clicks. In [14], the authors introduce a solution to the issue of traffic congestion, which is particularly problematic in Egypt and causes dangerous accidents and delayed traffic. He provides us with a straightforward plan to solve these issues using current technology like the Internet of Things. The traffic intersection is continuously shot by the camera, which records real-time photographs of it. Wi-Fi is used to transmit density data to the M2x IoT cloud platform (Server). A desktop Java program set up to monitor and control traffic lights through accessible data. The embedded device, which consists of a time relay to regulate traffic signal times, is connected to the control unit through a Raspberry Pi.

In order to save fuel and travel time, [15] provided an eco-driving system that maximized vehicle speed at signalized junctions. It learned from driving data and traffic signals using a Bayesian network and Gaussian process model to recommend the best speed modifications to drivers. Travel time and fuel efficiency have significantly improved, according to simulations. Additionally, to estimate traffic density in the event of sensor failures, [16] suggested using an adaptive-R extended Kalman filter (AREKF) in conjunction with a data imputation technique. The findings of the analysis and simulation demonstrated that AREKF enhances traffic flow via real-time ramp metering, hence lowering congestion, and estimated traffic density with unknown noise covariance with accuracy. Furthermore, In order to enhance traffic flow, [17] presented an MPC-based car-following method that makes use of V2V communication. It anticipated the future states of the vehicle in front of it and adjusts the control to reduce speed variations and dangerous gaps. Comparing it to current techniques, simulations revealed that it improves traffic flow at crossings.

It is worth also mentioned that to enhance traffic flow, [18] provided a roundabout control system (RCS) for automated and networked vehicles. Compared to previous systems, the RCS improved velocity, fuel efficiency, and travel time by clustering incoming cars and timing their merging. Additionally, A cyber-physical architecture for coordinating automated and networked cars on motorways is presented in [19]. In comparison to conventional systems, it improves fuel efficiency, velocity, and travel time by optimising vehicle trajectories for seamless lane changes and merging. In addition, [20] presented a cloud-based system for coordinating automated vehicles at roundabouts, using a two-level approach to optimize merging and minimize delays. Simulations showed it improved traffic flow compared to traditional systems.

Moreover, different models and algorithms have been proposed in the literature in order to enhance the connectivity of IoT network. An IoT network that uses Massive MIMO and Non-Orthogonal Multiple Access (NOMA) for B5G communications that is energy-efficient was proposed in [21]. Its goal is to make it possible for several scattered users and Internet of Things devices to communicate and transmit data seamlessly in order to improve energy efficiency and quality of service. The IoT connectivity in unlicensed spectrum for indoor smart home use-cases was explored in [22]. This study looked into two possibilities for channel access management: uncoordinated access, in which sensors and gateways function independently, and coordinated access, in which both synchronise their activity patterns. Also, in order to improve IoT connectivity, the study presented a distributed coordinating approach utilising reinforcement learning.

A low-power non-orthogonal multiple access beamforming (NOMA-BF) system for 6G wireless communication based on intelligent reflective surfaces (IRS) proposed in [23]. The optimization approach consists of two steps: the first uses user clustering and zero-forcing beamforming, while the second phase addresses passive beamforming at the IRS utilizing techniques. This proposed model is appropriate for modern 6G wireless communications since it accomplished efficient convergence and provides great energy efficiency performance with minimal computational complexity in the IRS-assisted NOMA-BF system. The integration of non-orthogonal multiple access (NOMA) into vehicle-to-everything (V2X) communications for beyond 5G transportation systems, aiming to improve traffic efficiency and reliability [24]. V2X communication, which involves highly mobile vehicles, faces challenges due to imperfect channel state information (CSI). The paper introduces an energy-efficient power allocation scheme for road-side units (RSUs) in cellular V2X networks, addressing outage probability, quality of service (QoS), and power constraints under the influence of imperfect CSI.

Additionally, ambient Backscatter Communication (AmBC) and Non-Orthogonal Multiple Access (NOMA) integration suggested in [25] to allow low-power Internet-of-Vehicles (IoVs) to be connected in upcoming 6G transportation systems. It presented an energy-efficient strategy for allocating resources to this NOMA IoV network with AmBC enabled, taking into account the difficulties associated with inaccurate Successive Interference Cancellation (SIC) decoding. The advantages of non-orthogonal multiple access (NOMA) and addresses the challenge of random collisions in wireless communication systems presented have been discussed in [26]. It improves power-domain multiple access (PDMA) methods by enhancing existing NOMA irregular repetition SA and introducing NOMA coded SA. However, PDMA-based methods require accurate sensing and power control, which may not be suitable for low-cost devices.

In particular, efficient and secure communication proposed in [27] vehicular networks that facilitate autonomous vehicle communication (AV2X), which are autonomous intelligent transportation systems. In order to notify emergency vehicles, ease traffic congestion, and help in low visibility circumstances, effective communication is essential. In this paper, an adaptive AV2X model based on optimisation methods and distributed deep learning approach is introduced to improve vehicular network connectivity. Moreover, [28] focused on improving traffic performance and road safety through vehicle-to-vehicle (V2V) communication. It tackles the problem of emergency vehicle response times in congested areas, especially in areas without designated emergency lanes. The suggested emergency vehicle route-clarifying technique makes use of vehicle-to-vehicle (V2V) communication to locate the closest vehicle, hence facilitating the emergency vehicle’s swift arrival at its destination. Additionally, the significance of effective communication in intelligent transportation systems that are autonomous, especially for autonomous vehicles that are connected to everything (AV2X) has been tackled in [29]. A novel optimization technique is used to improve vehicular network connectivity through the introduction of an adaptive AV2X model. This concept facilitates communication with autonomous vehicles (AVs) or information relaying to several entities by optimising inter-vehicle location.

Nonetheless various models have been proposed in the literature to tackle traffic management issues, an aspect of the effective application of IoT communication remains unexplored. The capacity to provide dependable connectivity between IoT devices and several stakeholders—including drivers and traffic authorities—represents this unrealized promise. The novel method to traffic management presented in this study highlights the vital requirement for trustworthy communication between IoT devices and all pertinent stakeholders, including the traffic control department and drivers. Improving road conditions, decreasing power, fuel consumption, and supporting an enhanced traffic management system are the main goals of this innovative approach.

## Proposed model

**Fig 1:**
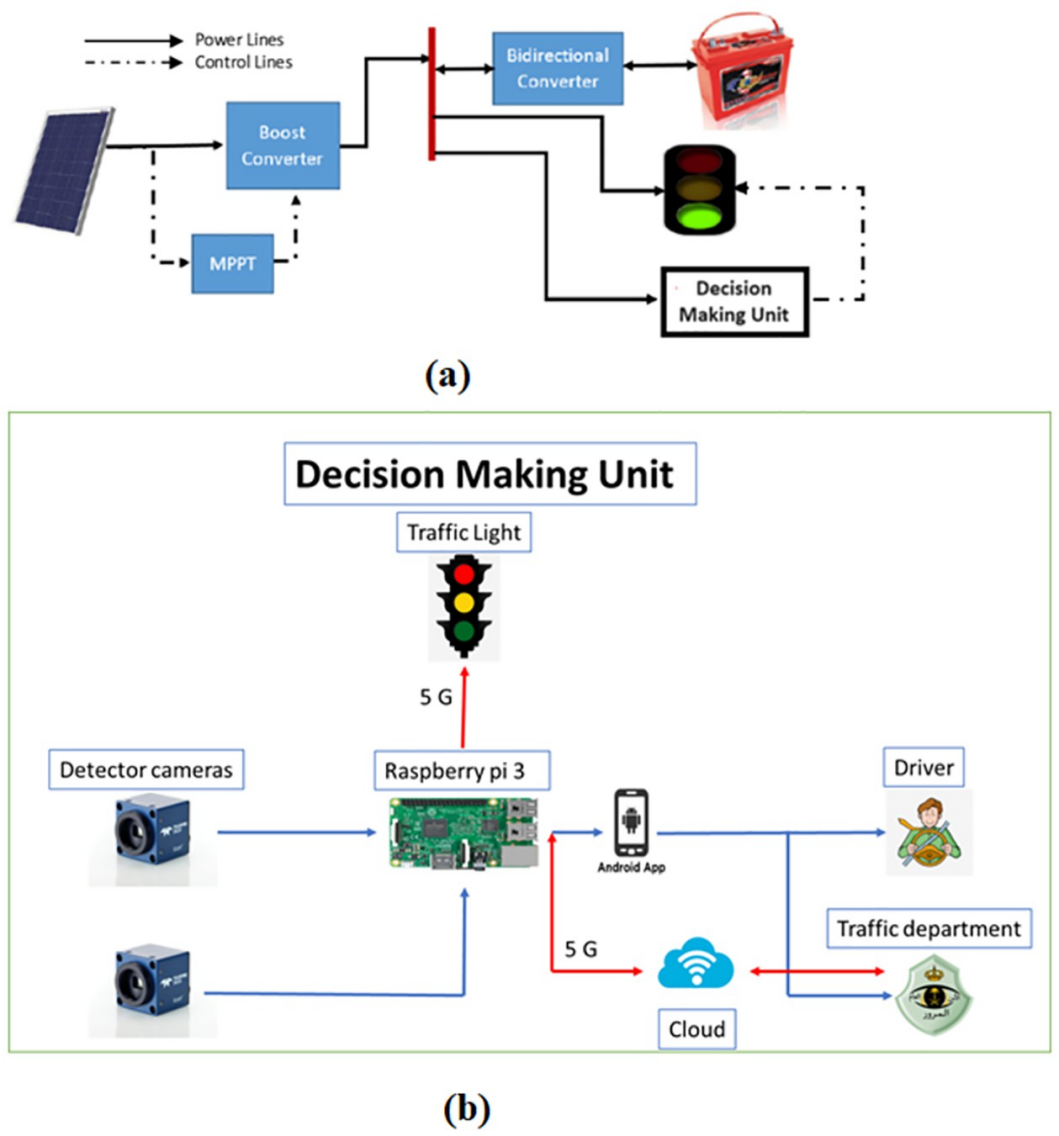
Proposed power and control system: (a) the PV powering system (b) the decision making control unit details.

**Fig 2:**
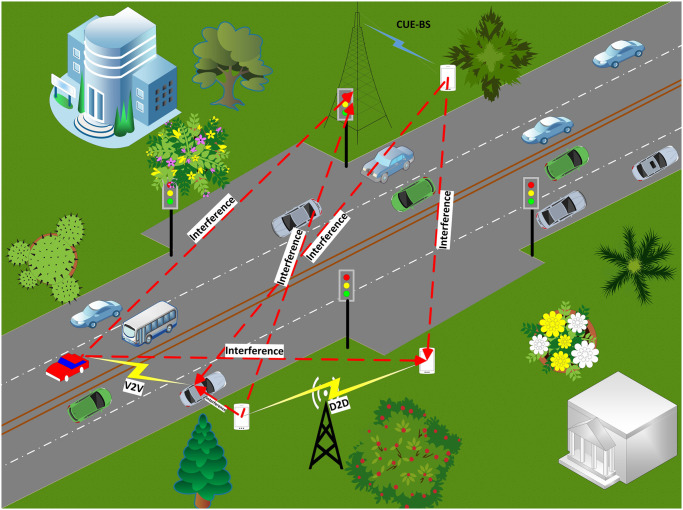
Proposed IoT communication system.

The significant issue of traffic congestion by leveraging the capabilities of IoT and 5G technologies is the focus of the proposed model. The objective is to mitigate air pollution, decrease fuel consumption, and enable timely arrival at destinations for car owners and at the same time ensure that IoT can communicate effectively with everything. The proposed traffic management system, illustrated in Figs 1 and 2, comprises two main parts where Fig 1a dedicated to the PV powering system Fig 1b depicts the decision making control unit details, while Fig 2 dedicated to the IoT communication system.

- Power system consists of: PV module, charger, Battery and traditional traffic light.
- Control system consists of: Detector cameras, raspberry pi 3 as micro controller, android app, cloud.
- The second part of the proposed model is IoT communication and how IoT communication can suffer from different type of interference.
- The interference may occur due to the existence of various transmitted devices a shown in Fig 2 such as the existence of cellular user equipment which communicate with base station and also the existence of transmitted devices (Dtx) which communicate with other device (Drx) this known as D2D communication.

In fear of fuel depletion the traffic light system is supplied using an auto clean solar powering system that directly power both the traffic light system as well as the backup battery during the daytime. In contrast, the battery takes the lead to power the traffic light system at night in order to provide clean energy and save the traditional power generation methods. From a control perspective, the cameras will identify the road and log data onto the microcontroller. This data will be processed using image processing techniques, and the results will be transmitted to both the cloud and an Android app. Fines and road-related information will be stored on the cloud server, accessible through the traffic department. The Android app will then display this information to the driver, providing updates on road conditions and any fines incurred.

### PV modelling and MPPT

**Fig 3:**
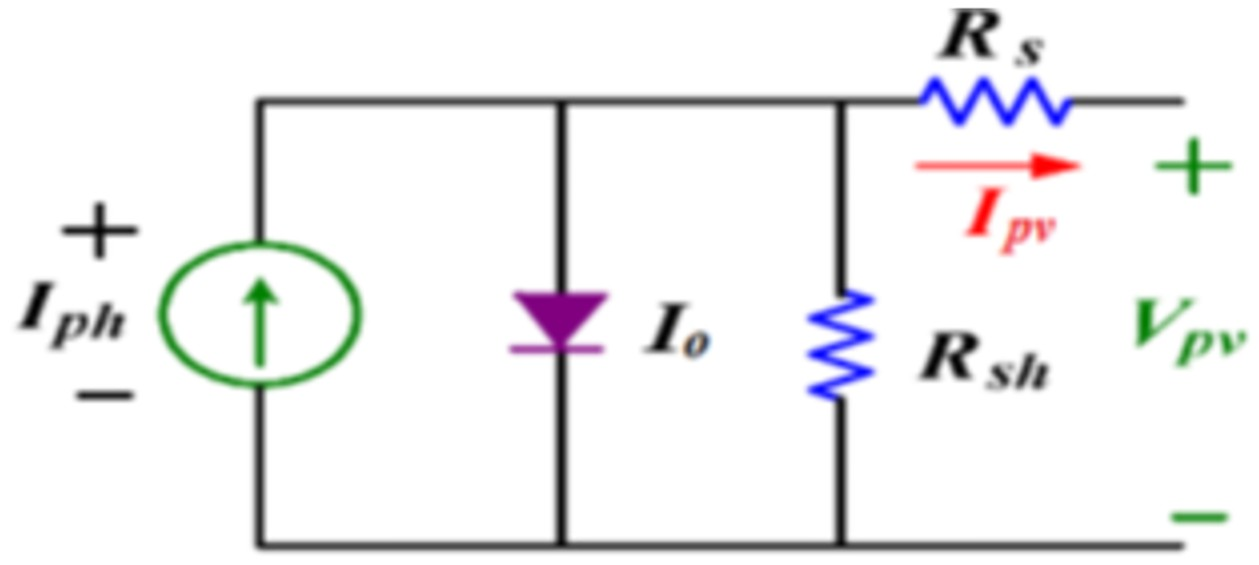
Single diode equivalent circuit of a solar cell.

The single-diode equivalent circuit illustrated in Fig 3, is deemed to be the basic electrical equivalent circuit of a solar cell. The relation between the PV module voltage *V*_*pv*_ and current *I*_*pv*_ referred to this model can be indicated as [30]

$$\begin{array}{c} I_{pv}=I_{ph}-I_{0}[\text{exp}\;(\frac{q(V_{pv}+I_{pv}R_{s})}{nkT})-1]-\frac{V_{pv}+I_{pv}R_{s}}{R_{sh}}. \end{array}$$ (1)

where, *V*_*pv*_ and *I*_*pv*_ represent the output voltage (V) and current (A) of the PV module, *I*_*ph*_ denotes the light current generated at a specific solar irradiance, Io is the diode reverse saturation current, q represents the electronic charge = 1.6*x*10^−19^C, n is the dimensionless deviation factor, *k* refers to Boltzmann’s constant = 1.3807*x*10^−23^*JK*^−1^, T shows the temperature of the PV cell (K), *R*_*s*_ and *R*_*sh*_ are the series and shunt resistances (Ω) that expresses the internal losses and the leakage current to ground respectively. Consequently, The (*V* − *I*) characteristic of a module with series and/or parallel cells can be expressed as follows in the up coming equation:

$$\begin{array}{c} I_{pv}^{M}=n_{p}I_{ph}-n_{p}I_{0}[\text{exp}\;(\frac{\left(\left(V_{pv}^{M}/n_{s}\right)+\left(I_{pv}^{M}R_{s}/n_{p}\right)\right)}{AV_{t}})-1]-\frac{\left(n_{p}/n_{s}\right)V_{pv}^{M}+I_{pv}^{M}R_{s}}{R_{sh}}. \end{array}$$ (2)

where, *n*_*p*_ denotes the shunt cells number and *n*_*s*_ denotes the series cells number in a PV module. It can be concluded from previous equations that the PV module output voltage and current are significantly affected by temperature and solar irradiance intensity. In turn the maximum power point is deviating under various environmental conditions. Thus, it is mandatory to track the maximum power point (MPP) continuously in order to harvest the PV system extracted power. In the current design, a boost type DC/DC converter is utilized to match the load to the PV solar system. Thus, extract the maximum power.

MPPT is an electronic system that varies the electrical operating point of the PV module to force it deliver its maximum available power. A modified P&O algorithm has been used due to its simple structure and few number of required measured parameters (*V*_*pv*_ and *I*_*pv*_). Hence, the corresponding power is mathematically calculated. It operates by forcing the module terminal voltage and current to make a small active perturbation then referenced to the previous calculated power. In case of power surplus, the perturbation continuous in the same direction: if else, the perturbation direction is reversed [31].

### DC/DC boost converter

**Fig 4:**
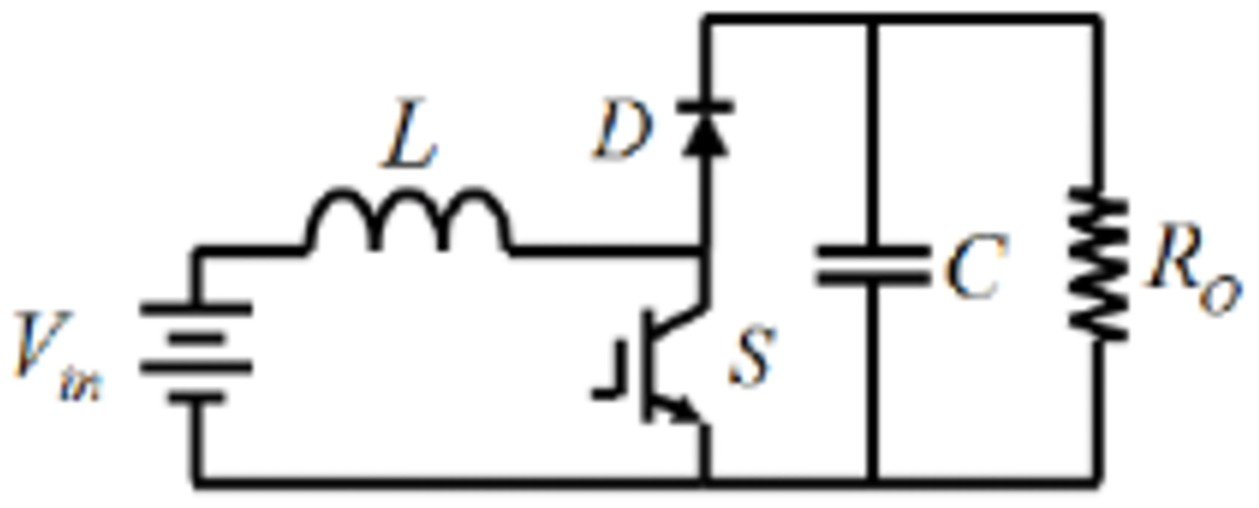
DC/DC boost converter schematic diagram.

The DC/DC boost power converter is known to be a step-up converter that plays a fundamental role in varying the voltage level to continuously track the MPP where the ratio between its output voltage (*v*_*o*_) and its input voltage (*v*_*i*_) is controlled by the duty cycle (D) of the switch. The schematic of Boost DC/DC converter is demonstrated in Fig 4 [32].

Having analysis about output voltage of this converter will be represented as shown in the following equation. Where the duty cycle D is defined as the time relationship that the switch is on relative to the total switching period. Assuming that all inductors’ current and capacitors’ voltage are kept constant, as normally in the switching converters’ steady state analysis, the DC voltage in the inductor *V*_*L*_ can be expressed as (3) and is equal to zero:

$$\begin{array}{c} V_{L}=D\left(V_{in}\right)-\left(1-D\right)\left(V_{in}-V_{o}\right)=0 \end{array}$$ (3)

From (3) at steady state in the ideal converter model, it can be verified that the capacitor’s voltage (output voltage) becomes:

$$\begin{array}{c} \frac{V_{o}}{V_{\text{in}}}=\frac{1}{1-D} \end{array}$$ (4)

The DC inductor’s current can be obtained by the input and output power:

$$\begin{array}{c} V_{in}I_{L}=V_{o}I_{out}=\frac{V_{o}V_{o}}{R_{o}} \end{array}$$ (5)

$$\begin{array}{c} I_{L}=\frac{V_{o}}{V_{in}}\frac{V_{o}}{R_{o}} \end{array}$$ (6)

Substituting (4) into (6), the inductor current can be expressed as:

$$\begin{array}{c} I_{L}=\frac{V_{o}}{\left(1-D\right)R_{o}} \end{array}$$ (7)

### Battery energy storage system

In a completely clean powering system, the ESS coupled to the DC bus is a common approach to compensate for the fluctuating nature of PV renewable energy supply. It promotes local power generation while ensuring a smooth and uninterrupted power flow to the load. Batteries and super-capacitors are the most prevalent and cost-effective energy storage devices in the low and medium-power range. In the examined application, a Lead-Acid BESS is used [33]. A battery state is primarily characterized by two important quantities, the battery terminal voltage (*V*_*b*_) and its state of charge (SOC), which are as follows [34]:

$$\begin{array}{c} V_{b}=V_{0}+R_{b}\cdot i_{b}-\frac{KQ}{Q+\int i_{d}\;dt}+A\;\text{exp}\;(B\int i_{d}\;dt) \end{array}$$ (8)

$$\begin{array}{c} SOC=100(1+\frac{\int i_{d}\;dt}{Q}) \end{array}$$ (9)

where *V*_*o*_ is the open circuit voltage of the battery, *R*_*b*_ is the internal resistance of the battery, *i*_*b*_ and *Q* are the charging current and capacity of the battery, respectively, *K* is the polarization voltage, *A* is exponential voltage, and *B* is exponential capacity. If one of the most significant futures is an advanced framework that may permit the connectivity of diverse generation systems, energy storage alternatives, and various loads with optimal asset utilization and operation efficiency. These objectives can be met with the help of power electronics technology, which is critical for connecting various sources and loads [35]. As a result, a BESS is linked to a bidirectional DC/DC converter, which allows power to be transferred to and from the battery.

### Bidirectional DC/DC converter

**Fig 5:**
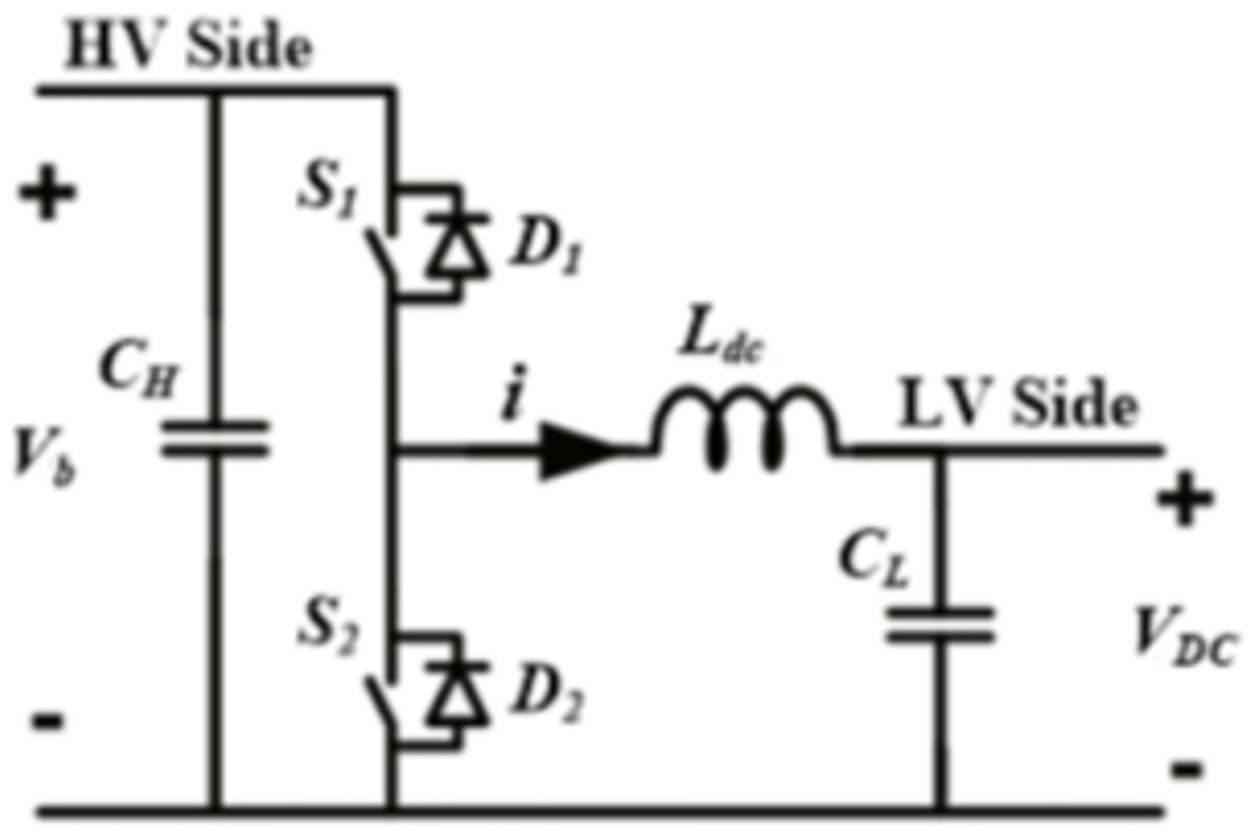
DC/DC bidirectional converter schematic diagram.

A basic buck or boost converter can be converted into a bidirectional converter by replacing the diodes that prohibit reverse current flow in their structure with a programmable switch, resulting in the identical structure in both cases, as illustrated in Fig 5 [36]. It is possible to deduce that the fundamental non-isolated bidirectional DC/DC converter is a mix of antiparallel step-up and step-down stages [37]. As a result, depending on where the additional energy storage is placed, the resulting converter might be classified as buck or boost. The converter is classed as buck if the ESS is located on the high voltage side (HVS), as in the proposed configuration, and boost if it is placed on the low voltage side (LVS) [38].

**Fig 6:**
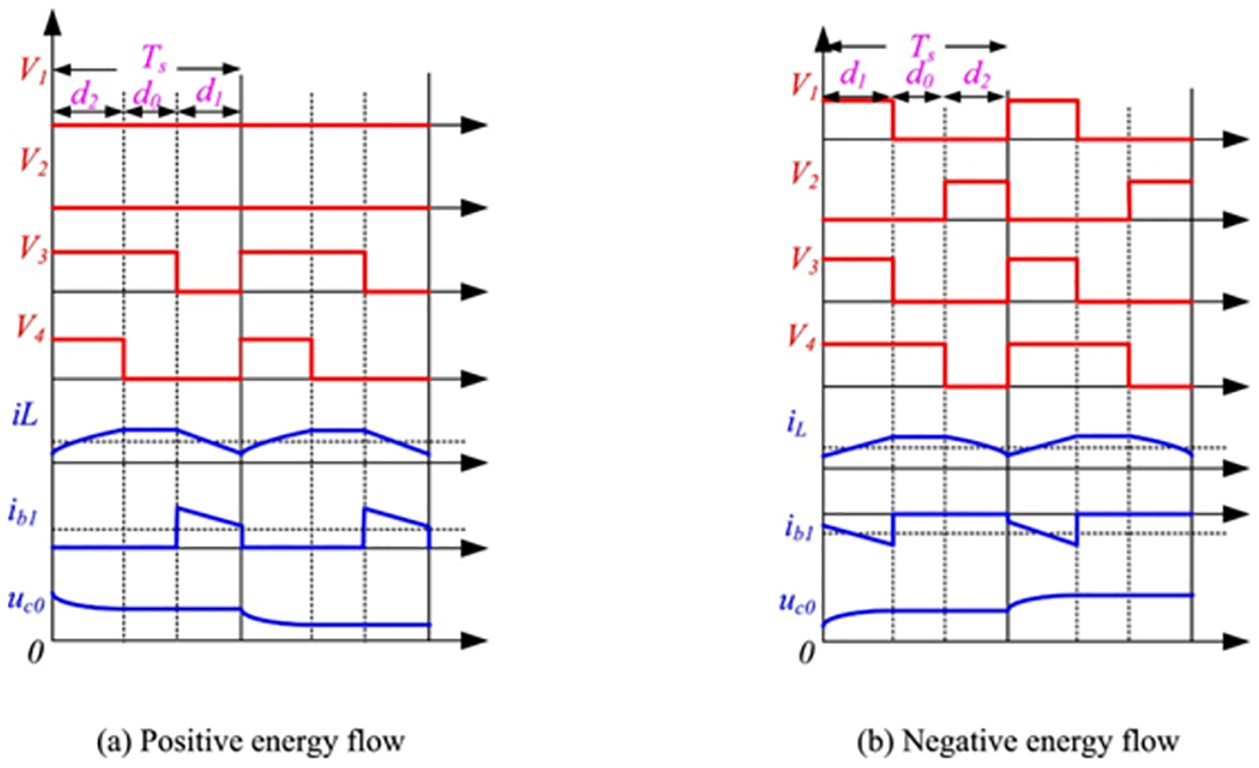
Waveforms of the Bidirectional DC/DC Converter: (a) positive energy flow (b) negative energy flow.

Power is transferred in either buck or boost mode depending on how the switches (*S*_1_, *S*_2_) are switched in conjunction with their anti-parallel diodes (*D*_1_, *D*_2_). Fig 6 shows the basic waveforms associated with the bidirectional DC/DC buck converter’s main circuit when the BESS terminal voltage (*V*_*b*_) is larger than the DC bus voltage (VDC). In buck mode, power is transferred from the HVS to the LVS; in this case, *S*_1_ works as an active regulated switch while *S*_2_ is turned off, and the inductor (*L*_*dc*_) is employed as a low-pass filter, resulting in lesser ripple current when the BESS is discharging.

$$\begin{array}{c} D=\frac{V_{DC}}{V_{b}} \end{array}$$ (10)

In boost mode, power is transferred from the LVS to the HVS, with *S*_2_ in an active regulated state and *S*_1_ turned off, while the *L*_*dc*_ serves as a boost inductor and the BESS charges [38].

$$\begin{array}{c} D=1-\frac{V_{DC}}{V_{b}} \end{array}$$ (11)

where *D* denotes the duty cycle and is defined as the ratio of the time when *S*_1_ is turned ON to the switching period *T*. Furthermore, both *C*_*H*_ and *C*_*L*_ function as energy buffers.

### Proposed IoT communication

For the proposed IoT communication network, it is assumed that there are *I* number of IoT devices, *C* number of cellular user equipment (CUE), *L* number of D2D links and *V* number of V2V links. the communication between IoT devices and the drivers or the traffic management and the interference that may occur due to sharing the CUE, D2D links and V2V links the same spectrum with IoT network is shown in Fig 2. Therefore, the suggested approach seeks to improve the performance of IoT communication by optimizing the network’s total energy efficiency (*EE*), which can be stated as follows:

$$\begin{array}{c} \text{max}\;\sum_{c=1}^{C}\sum_{d=1}^{D}\sum_{v=1}^{V}{EE}_{c,d,v},\;{EE}_{c,d,v}≔f_{1}\left(P_{I},SINR_{IoT}\right). \end{array}$$ (12)

subject to

$$\begin{array}{cc} P_{I} & \leq P_{Imax},\\ SINR_{IoT} & \geq SINR_{th} \end{array}$$

where *EE*_(*C*,*D*,*V*)_ represents the *i* − *th* path’s system energy efficiency between an IoT device, drivers, and traffic management. The signal-to-interference-plus-noise ratio required by the system and for IoT to drivers and traffic management are represented by the symbols *SINR*_*th*_ and *SINR*_*IoT*_, respectively. The IoT transmission power and the maximum IoT transmission power are represented by the symbols *P*_*I*_ and *P*_*Imax*_, respectively.

Non-orthogonal multiple access (NOMA) is therefore regarded as the suitable access approach for the suggested model in order to serve the IoT devices and permit them to access the channel at the same frequency or at the same time [39, 40]. Furthermore, the suggested model assumes an additive white Gaussian noise (AWGN) Rayleigh fading channel [41]. Moreover, statistical mutual independence between the channel fading coefficients for different transmission links is assumed. Therefore, the IoT network energy efficiency (*EE*) can be expressed as:

$$\begin{array}{c} EE_{IoT}=\frac{R_{IoT}}{P_{I}+P_{o}}. \end{array}$$ (13)

$$\begin{array}{c} EE_{IoT}=\frac{B}{P_{I}+P_{o}}\;{\text{log}}_{2}\;(1+\frac{P_{I}H_{IX}}{\sum_{k=1}^{K}P_{C}H_{C_{k}X}+\sum_{l=1}^{L}P_{D}H_{D_{l}X}+\sum_{v=1}^{V}P_{v}H_{V_{v}X}+N}). \end{array}$$ (14)

where *R*_*IoT*_, *P*_*I*_ and *P*_*o*_ are the achievable data of IoT devices, IoT transmission power respectively and the internal circuitry power consumption, respectively. $H_{I_{X}}$, $H_{C_{k}X}$, $H_{D_{l}X}$ and $H_{V_{v}X}$ are the channel gain coefficient between IoT and everything whether it was the driver or the traffic management, between any cellular user equipment (CUE) and everything, any transmitted device (Dtx) and everything, and any transmitted vehicles (Vtx) and everything respectively. *P*_*C*_, *P*_*D*_ and *P*_*V*_ are the CUE, Dtx and Vtx interference transmission power, respectively. *B* and *N* are channel system bandwidth and the noise power, respectively.

Eq (12) illustrates the primary objective of the proposed strategy, which is to maximize total energy efficiency (*EE*) under different environmental conditions. Thus, the optimization problem provided in (12) has the following Lagrangian, which may be expressed as follows:

$$\begin{array}{c} L\{PI,{SINR}_{IoT},\lambda_{1},\lambda_{2}\}=EE+\lambda_{1}\left(P_{Imax}-P_{I}\right)+\lambda_{2}\left(SINR_{th}-SINR_{IoT}\right) \end{array}$$ (15)

where λ_1_ and λ_2_ are the non-negative Lagrangian multipliers. By considering the derivative of Eq (15) with respect to *P*_*I*_ and Pint, it is able to calculate the values of λ_1_ and λ_2_ for satisfying the constraint of the optimisation problem for (*EE*). As a result, λ_1_ and λ_2_ are as follows:

$$\begin{array}{ccc} \lambda_{1}=-\frac{B}{{\left(P_{I}+P_{o}\right)}^{2}}\;{\text{log}}_{2}\;(1+\frac{P_{I}H_{IX}}{\sum_{c=1}^{C}P_{C}H_{C_{c}X}+\sum_{d=1}^{D}P_{D}H_{D_{d}X}+\sum_{v=1}^{V}P_{v}H_{V_{v}X}+N}) & & \\ +(\frac{B}{\left(P_{I}+P_{o}\right)}\frac{\sum_{c=1}^{C}P_{C}H_{C_{k}X}+\sum_{d=1}^{D}P_{D}H_{D_{l}X}+\sum_{v=1}^{V}P_{v}H_{V_{v}X}+N}{P_{I}H_{IX}+\sum_{c=1}^{C}P_{C}H_{C_{k}X}+\sum_{d=1}^{L}P_{D}H_{D_{l}X}+\sum_{v=1}^{V}P_{v}H_{V_{v}X}+N})*X+X*\lambda_{2} & & . \end{array}$$ (16)

where

$$\begin{array}{ccc} \;X=(\frac{H_{IX}}{\sum_{c=1}^{C}P_{C}H_{C_{c}X}+\sum_{d=1}^{D}P_{D}H_{D_{d}X}+\sum_{v=1}^{V}P_{v}H_{V_{v}X}+N}) & & . \end{array}$$

As described *P*_*C*_, *P*_*D*_ and *P*_*V*_ are the interference power of the transmitted CUE, *D*_*tx*_ and *V*_*TX*_. It has been assumed that all the transmitted interference devices are going to send their data with same interference power which is denoted by *P*_*int*_.

$$\begin{array}{ccc} & & \lambda_{2}=\frac{B}{\left(P_{I}+P_{o}\right)}(\frac{\sum_{c=1}^{C}P_{int}H_{C_{c}X}+\sum_{d=1}^{D}P_{int}H_{D_{d}X}+\sum_{v=1}^{V}P_{int}H_{V_{v}X}+N}{P_{I}H_{IX}+P_{int}(\sum_{c=1}^{C}H_{C_{c}X}+\sum_{d=1}^{D}H_{D_{d}X}+\sum_{v=1}^{V}H_{V_{v}X}+N)}) \end{array}$$ (17)

Moreover, by deriving Eq 15 with respect to λ_1_ and λ_2_, the optimal required IoT transmission power (*P*_*I*_) and the optimal required interference power (*P*_*int*_) for any interfering devices—including CUE, Dtx, and Vtx—can be determined as follows:

$$\begin{array}{c} P_{I}=P_{Imax} \end{array}$$ (18)

$$\begin{array}{ccc} & & P_{int}=\frac{P_{I}H_{IX}-N*SINR_{th}}{\;SINR_{th}(\sum_{c=1}^{C}H_{C_{c}X}+\sum_{d=1}^{D}H_{D_{d}X}+\sum_{v=1}^{V}H_{V_{v}X})} \end{array}$$ (19)

### Dataset generation

MATLAB simulations have been utilised to implement the equations of the proposed model, as detailed in Section, and provide the necessary datasets. To improve the IoT communication network, the datasets will be used to train models that will be put on all transmitting devices. The required signal-to-interference-plus-noise-ratio threshold (*SINR*_*th*_), the IoT device transmission power (*P*_*I*_), interference transmission power (*P*_*int*_), and the distances between IoT and everything (*d*_*IX*_), CUE and everything (*d*_*CX*_), Dtx and everything (*d*_*DX*_), and Vtx and everything (*d*_*VX*_) are all illustrated in each of the 21,744 records that have been generated.

**Fig 7:**
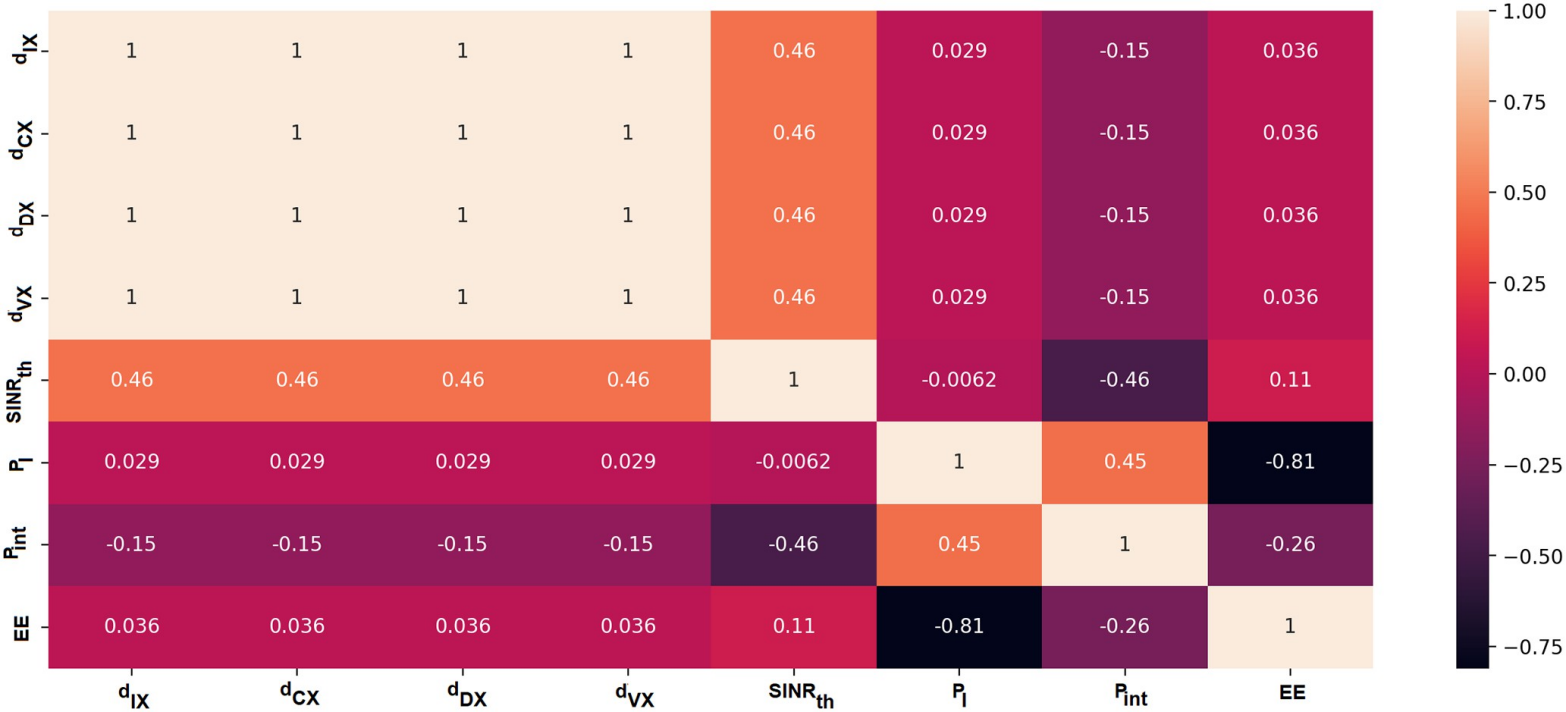
Pearson correlation coefficients of each input parameter (*d*_*IX*_, *d*_*CX*_, *d*_*DX*_, *d*_*VX*_, *SINR*_*th*_, *P*_*I*_) and the output (*P*_*int*_ and EE).

The relationship between each input and output parameter is shown by the Pearson coefficients in Fig 7. With the exception of the *P*_*I*_ parameter, which has a strong negative connection with EE, the graph shows that the output EE has a low correlation with all of the input values. Additionally, there is a strong negative association between the *SINR*_*th*_ parameter and parameters *P*_*int*_. Each of these factors must be taught to the deep learning model, and the results section will provide an explanation of the correlation’s impact.

### Proposed deep learning model

**Fig 8:**
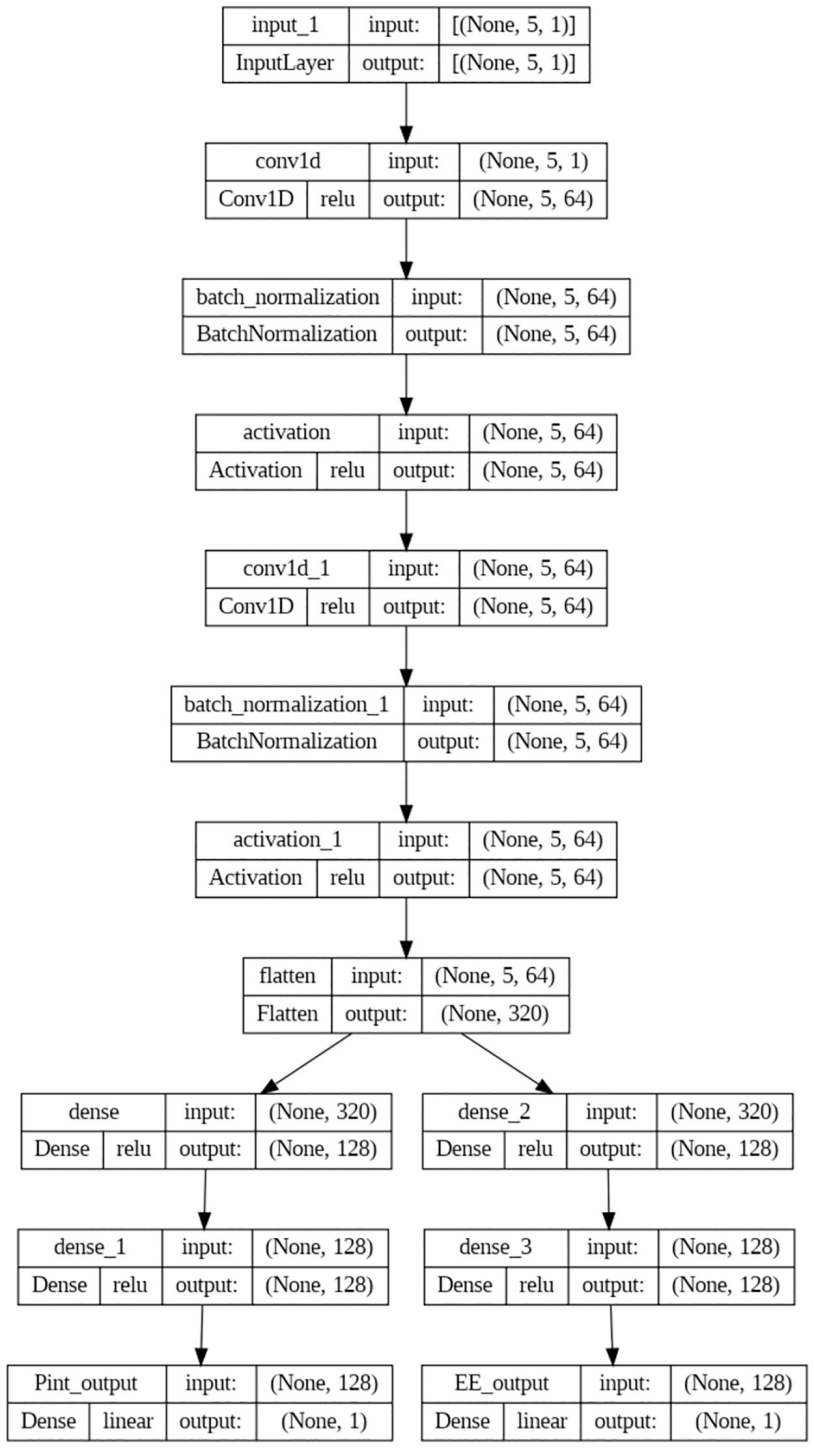
Proposed deep learning model.

The suggested deep learning model is put forth and explained in this section. To facilitate the learning of the model weights, a normalisation phase must be completed before the variables are entered into the suggested deep learning model. Prior to being added to the model, each variable is normalised using the min-max scaling technique. The five input variables *d*_*IX*_, *d*_*CX*_, *d*_*DX*_, *d*_*VX*_, *SINR*_*th*_, and *P*_*I*_ are used to determine the output parameters Pint and EE of the final dense layer. The model consists of three distinct phases: 1D-CNN, flattening, and thick layers, as illustrated in Fig 8. To process the normalized input parameters, three 1D-CNN layers—each with 64 filters and a size one kernel—are used.

For the purpose of reliably maintaining the breadth of the output matrix, each 1D-CNN layer produces padded data. Next, a flattening layer receives the output from the third 1D-CNN, reformats the dimension, and gets it ready to feed into the dense layers. The four dense layers that follow the flattening layer produce the regression result. The primary purpose of the deep learning (DL) model is to improve the IoT network’s performance by optimising crucial variables like transmission power and energy efficiency. The DL model specifically attempts to:

- Estimate Optimal Transmission Power (PI): Precisely estimate the transmission power needed by Internet of Things (IoT) devices to provide dependable connectivity while reducing interference.
- Enhance Energy Efficiency (EE): Evaluate and maximize the network’s energy efficiency to lower total power consumption and increase system sustainability.

A grid search was done to assess several combinations before deciding on the number of filters for the 1D-CNN and the number of nodes for the dense layers. The Rectified Linear Unit was used to activate each concealed layer (ReLU). In addition, the grid search involved testing out different approaches that might track the hidden layers in the proposed model while accounting for activation function selection. The best-performing parametric rectified linear unit (PReLU) activation function received the output from each hidden layer. The adaptive moment (Adam) is the optimization utilized in the suggested model, which has the mean absolute error (MAE) loss function and root mean squared error (RMSE) as targets. Adam may adaptively learn the proper parameters depending on the learning process. Whereas RMSE is the root square of the average of the squared differences between actual and anticipated values, it is in charge of evaluating the average difference between real and predicted values. This can be written as follows:

$$\begin{array}{c} MAE=\frac{\sum_{j=1}^{n}\left|y_{j}-x_{j}\right|}{n} \end{array}$$ (20)

$$\begin{array}{c} RMSE=\sqrt{\frac{\sum_{j=1}^{n}{\left(y_{j}-x_{j}\right)}^{2}}{n}} \end{array}$$ (21)

where *x*_*j*_ is the predicted value, *y*_*j*_ is the actual value, and *n* is the total number of recorded data points. The experiments conducted to train, test, and evaluate the proposed model are described in the section that follows.

A few difficulties arose during the deep learning model’s implementation, such as making sure there was enough good data available for the model’s training. The availability of precise and extensive datasets is critical to the model’s performance. Furthermore, Significant computational resources are needed for training deep learning models, which might be a drawback, particularly in contexts with limited resources. This includes the requirement for strong hardware and long training periods. Additionally, there is a chance that the model will become overfit to the training set, which could affect how well it generalises to new, untested data. To mitigate this issue, thorough tuning and regularisation approaches are required. We have produced a little amount of data to improve the diversity and robustness of the dataset in order to solve data restrictions. Additionally, dropout techniques and cross-validation to was implemented to reduce overfitting and enhance the model’s generalization skills. Thorough testing on a variety of datasets aids in confirming the performance of the model.

## Results and discussion

The whole proposed smart traffic management system has been simulated using several softwares. MATLAB/SIMULINK^®^ software package has been used for simulating both the PV system as power part of traffic system and the fuel consumption in the vehicles. While the Any Logic software has been simulated the control system part of the traffic system. While, for the communication system between IoT devices MATLAB and Python simulations were used to evaluate the effectiveness of the suggested strategy in terms of required interference power and optimized energy efficiency.

### PV simulation

**Fig 9:**
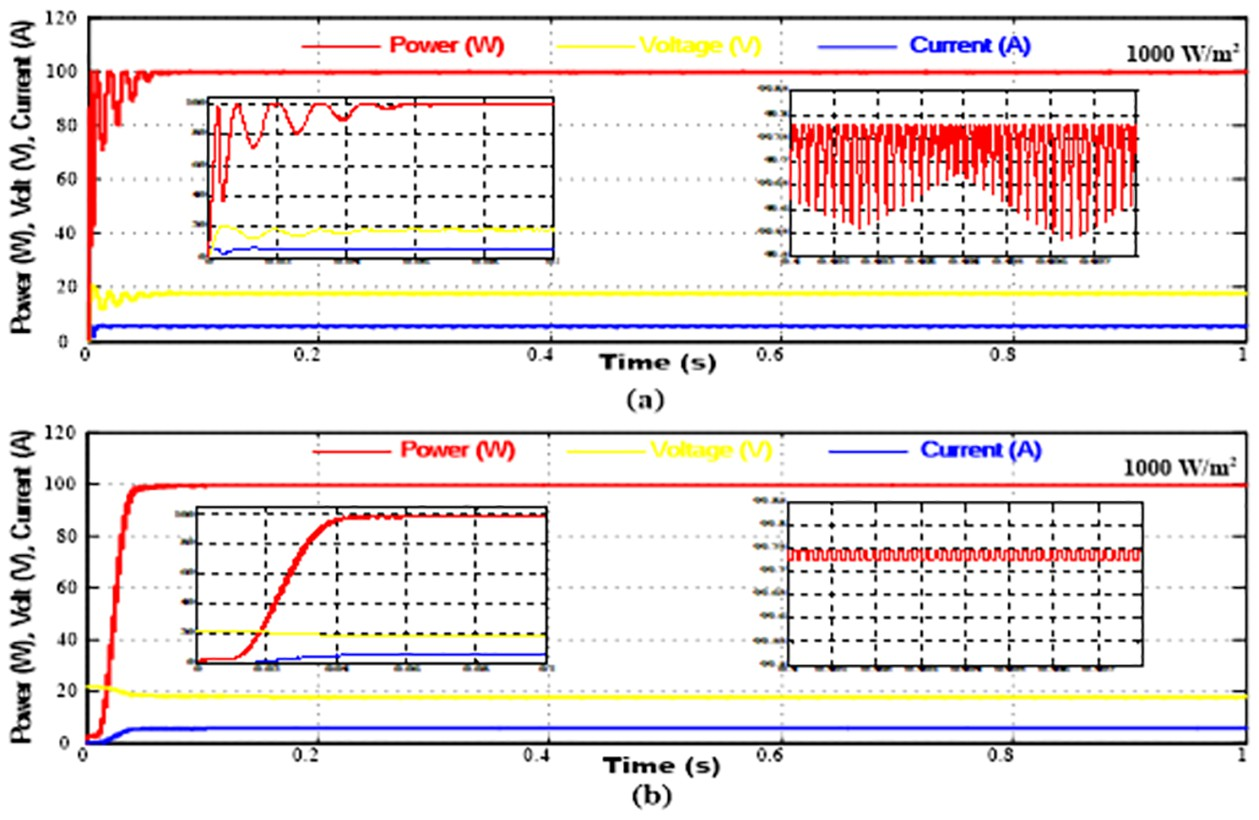
Steady-State Simulation Performance at 1000 *W*/*m*^2^ (a) Conventional P&O Algorithm, (b) Proposed SF Algorithm.

**Fig 10:**
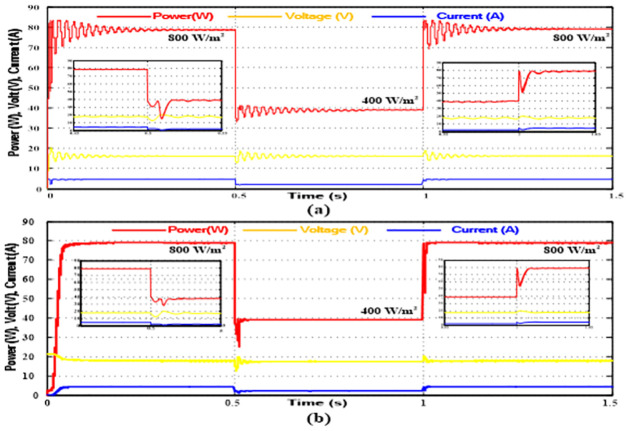
Transient Simulation Performance at 800*W*/*m*^2^ and 400*W*/*m*^2^ (a) Conventional P&O Algorithm, (b) Proposed SF Algorithm.

To verify the validity and practicality of the proposed MPPT method, it is simulated using the MATLAB/SIMULINK^®^ software package at a constant temperature of 25°*C* and rapid variations in solar irradiation levels. The results are compared with the results of the standard P&O approach. Tables 1 and 2, respectively, provide details on the simulated S405M36ULTRA PV panel and the DC/DC boost converter that was used to enhance the PV output voltage to the range of the battery voltage needed to power this system. Furthermore, by comparing the battery’s voltage with the load’s voltage, PI control is utilized to correct errors and regulate the battery’s charging and discharging using the bidirectional DC/DC converter. Figs 9 and 10. show the outcomes of the simulation.

**Table 1:** PV panel specifications.

Parameter	Value
Electrical characteristics	S405M36 Ultra
Open-circuit voltage (*V*_*OC*_)	21.5 V
Short-circuit current (*I*_*SC*_)	6.2 A
Voltage at maximum power	17.2 V
Current at maximum power	100 W
Maximum power	5.9 A
Temperature of *V*_*OC*_	-0.36% K
Temperature of *I*_*SC*_	0.03% K

**Table 2:** Parameters of DC/DC boost converter.

Parameter	Value
Switching frequency	5 kHz
Capacitor (*C*)	1000 *μ*F
Inductor (*L*)	2.5 mH
Inductor resistance	0.022 Ω
IGBT	STGP35HF60W
Fast recovery diode	BY329

Two scenarios were indicated. The first compares the new algorithm’s steady state and startup performance with the standard approach at solar irradiation levels of 1000*W*/*m*^2^ at a constant temperature of 25°*C*. The system’s transitory reaction to a step change in insulation level is depicted in the second scenario.

At a solar irradiation level of 1000 *W*/*m*^2^, Fig 9 demonstrates the effectiveness of the suggested algorithm in achieving the MPP of the PV system more quickly than the traditional method with less oscillations. The system transient reaction to a change in solar irradiation level from 800 *W*/*m*^2^ to 400 *W*/*m*^2^ at t = 0.5 s and back to 800 *W*/*m*^2^ at t = 1 s, while maintaining a constant temperature of 25°*C*, is compared between the two methods in Fig 10. When exposed to a step change in the solar irradiance level from 800 *W*/*m*^2^ to 400 *W*/*m*^2^, a system using the conventional algorithm depicted in Fig 10a takes tfall = 0.18 s to attain its new MPP and trise = 0.2 s to settle back at its initial MPP value when the solar irradiance level increases back to 800 *W*/*m*^2^. On the other hand, the system that uses the suggested SF MPPT algorithm, as seen in Fig 10b, has tfall = 0.09 s and trise = 0.08 s. It is clear from comparing the two methods’ performances that the suggested technique has a shorter transition time and produces less oscillations around the MPP.

### Smart traffic management system

**Fig 11:**
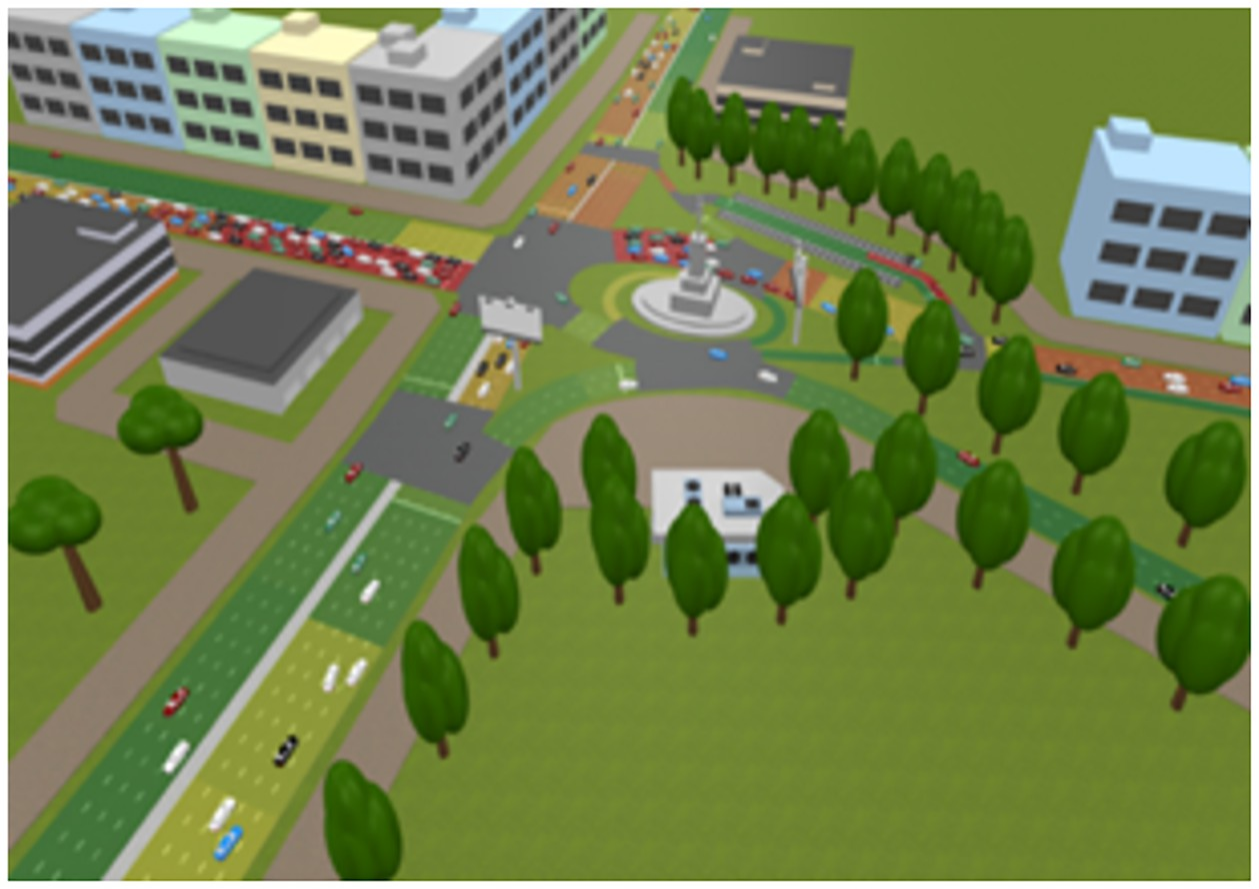
Smart traffic system simulation by any logic program.

**Fig 12:**
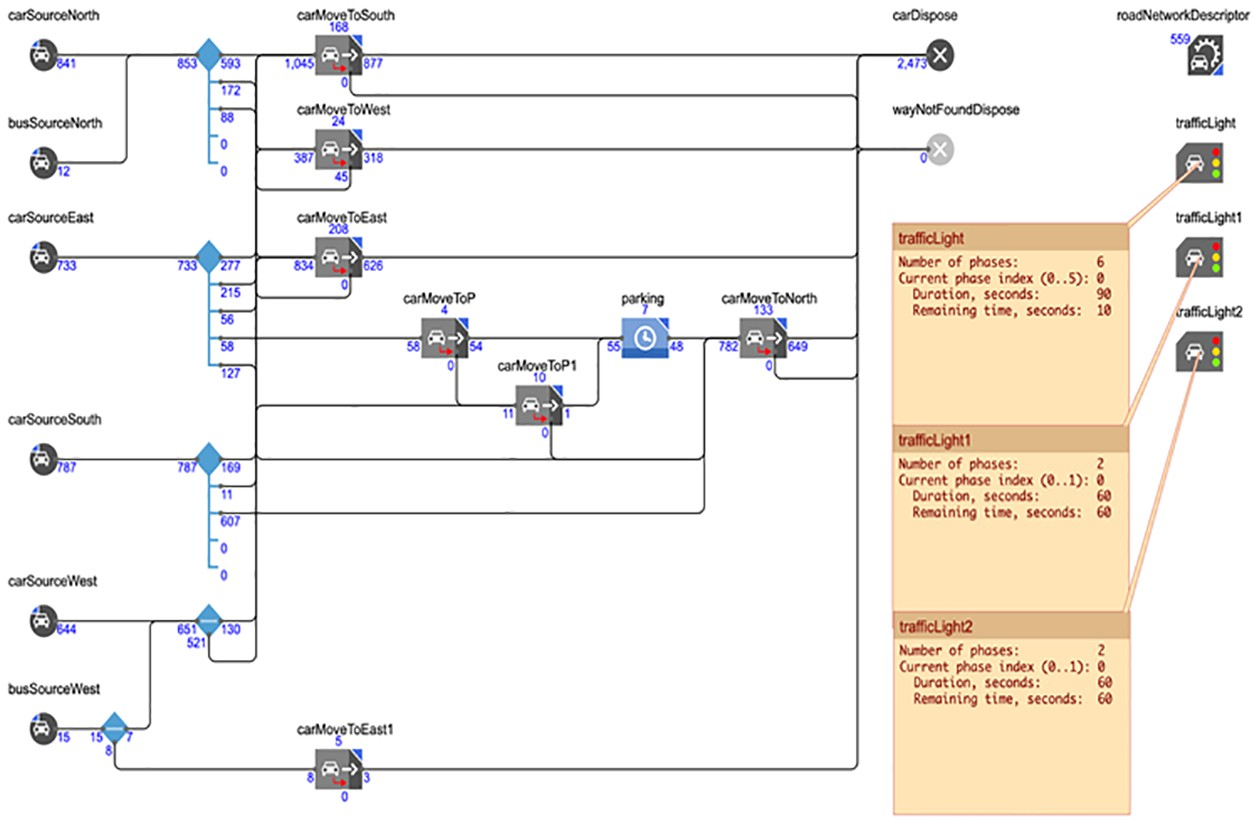
Smart traffic system simulation by any logic program.

Any logic program is used to simulate our suggested smart traffic management system’s control component. A proposed model for a management system to address the issues of traffic congestion and junctions is shown in Fig 11, which depicts the intended region. As seen in Fig 11, there are cameras above the stop lines that indicate the traffic. The logic circuit for the suggested simulation, in which we plan the traffic for this area, is depicted in Fig 12. There are several vehicles entering and leaving each traffic jam. The proposal is to install three traffic signals. Phases make to the design of traffic light number 1. To control traffic in this area, each phase is made for a distinct set of roads, some of which will be green and others of which will be red.

### Cars fuel consumption simulation

**Fig 13:**
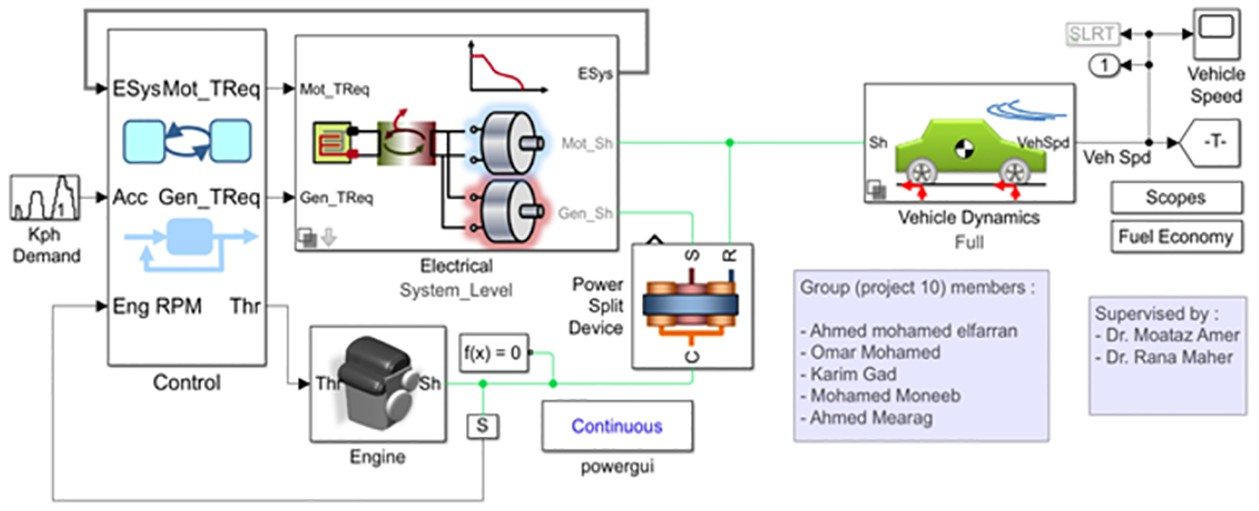
MATLAB Simulink for a standard vehicle.

**Fig 14:**
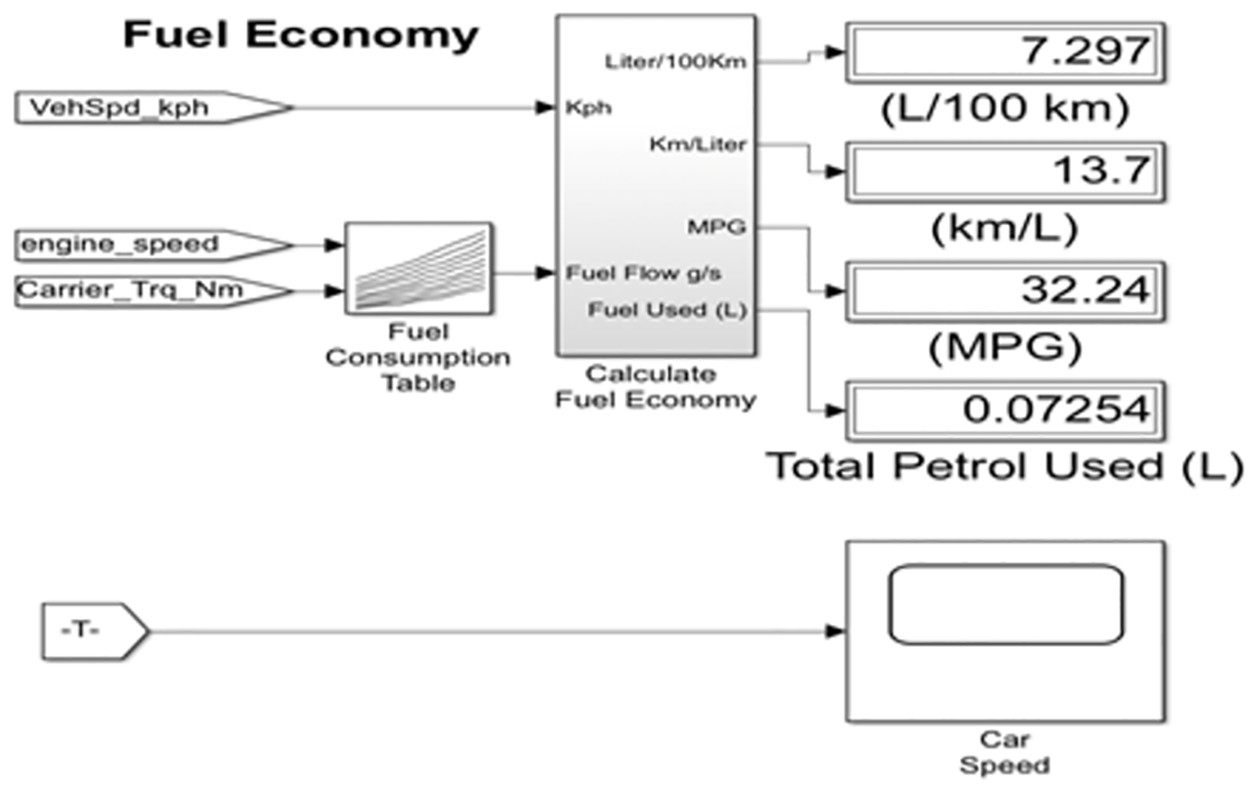
Fuel Economy by MATLAB Simulink.

The fuel consumption of the autos in the traffic is then calculated by combining the MATLAB/SIMULINK^®^ software package with Any Logic simulation. The mechanics and dynamics of the automobile are shown in Fig 13 below. The fuel efficiency typically determines how much gas the vehicle uses overall. Three inputs are used to calculate fuel economy: the vehicle speed in kilometers per hour, the engine speed, and the torque, as seen in Fig 14.

**Fig 15:**
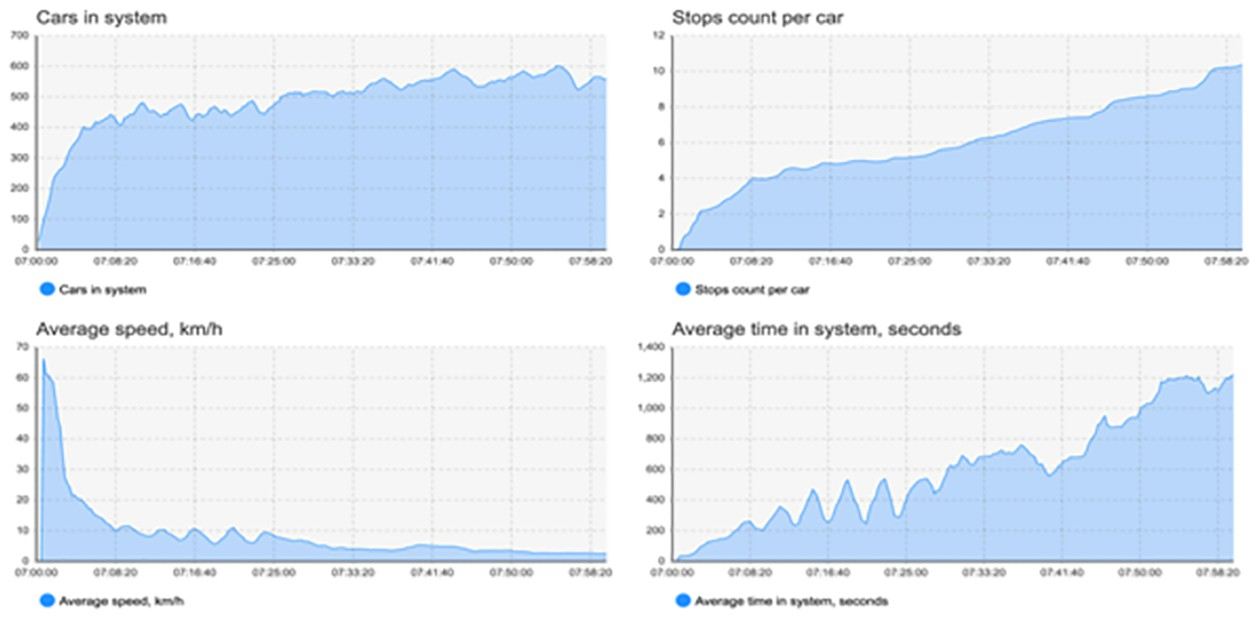
Statistics of any logic simulation.

**Fig 16:**
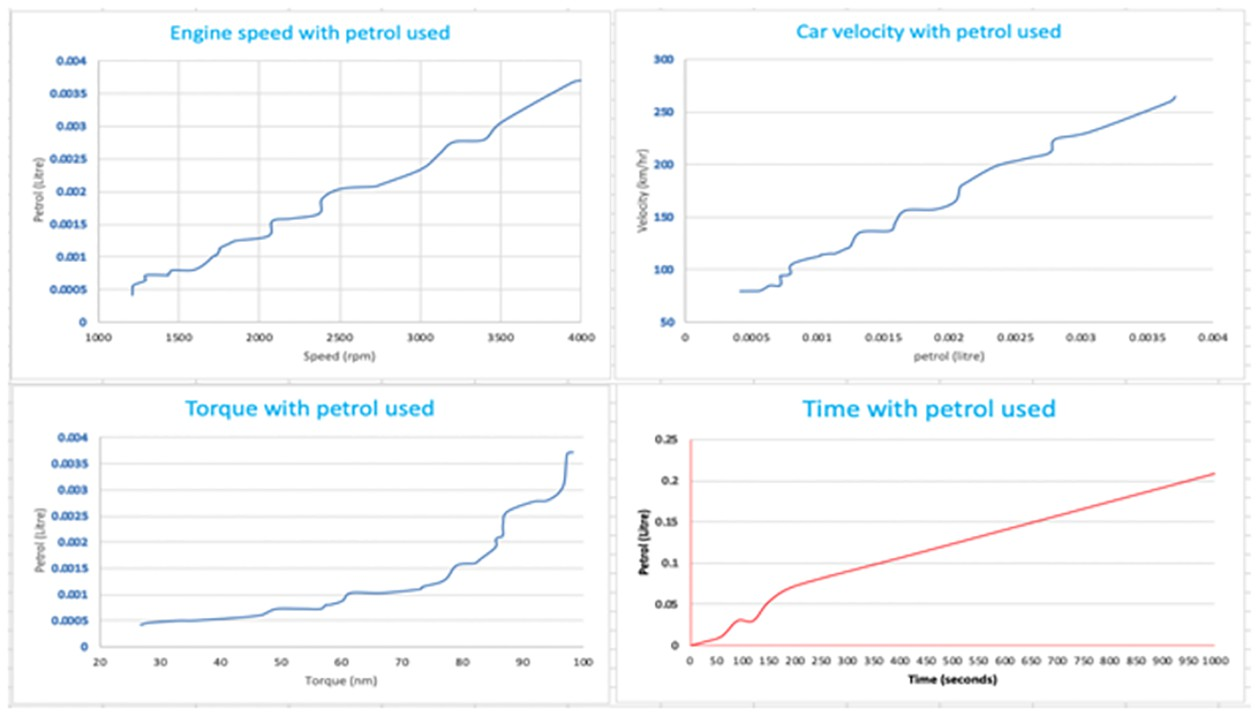
Curves of petrol used based on statistical data.

Fig 15 shows the curves that were produced by the Any logic simulation. The average speed of automobiles decreased and the average duration in the system grew as the number of cars and the number of stops per car increased. As seen in Fig 16, the curves produced by MATLAB/SIMULINK^®^ software package demonstrated the relationship between engine speed, vehicle velocity, torque, and time with gasoline utilized by the vehicle. By optimizing traffic signal timing, this suggested algorithm helps to lower traffic congestion and, as a result, fuel consumption.

### Simulation of the proposed IoT communication

**Fig 17:**
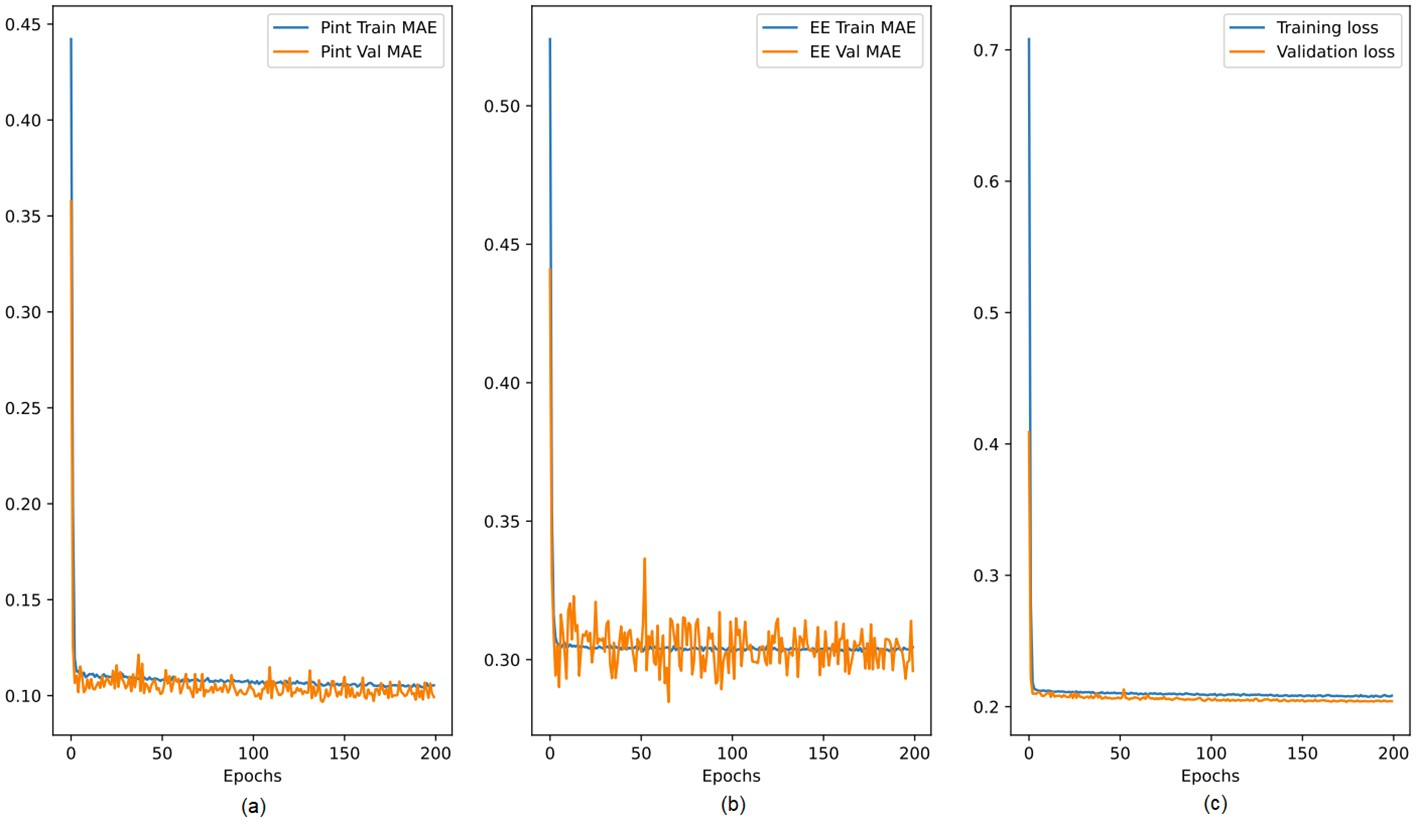
Training and validation mean absolute error generated during training the proposed model.

This section presents the performance of the suggested deep learning and analytical models. The testing and assessment of the suggested deep learning model, which is covered in Section, is shown in Fig 17. 80% of the datasets were used for training, while 20% were used for testing. The training and validation mean absolute errors for the required *P*_*int*_ and EE are shown in Fig 17a and 17b, respectively.

Since there was little change in the results after epoch 100, all of the numbers show that additional training was not necessary. The independent training and validation errors for each output were also near to the same values, as shown in Fig 17c, suggesting that the suggested model was neither overfitted nor underfitted. Additionally, the independent training and validation error losses decrease and are maintained at a specific point.

**Fig 18:**
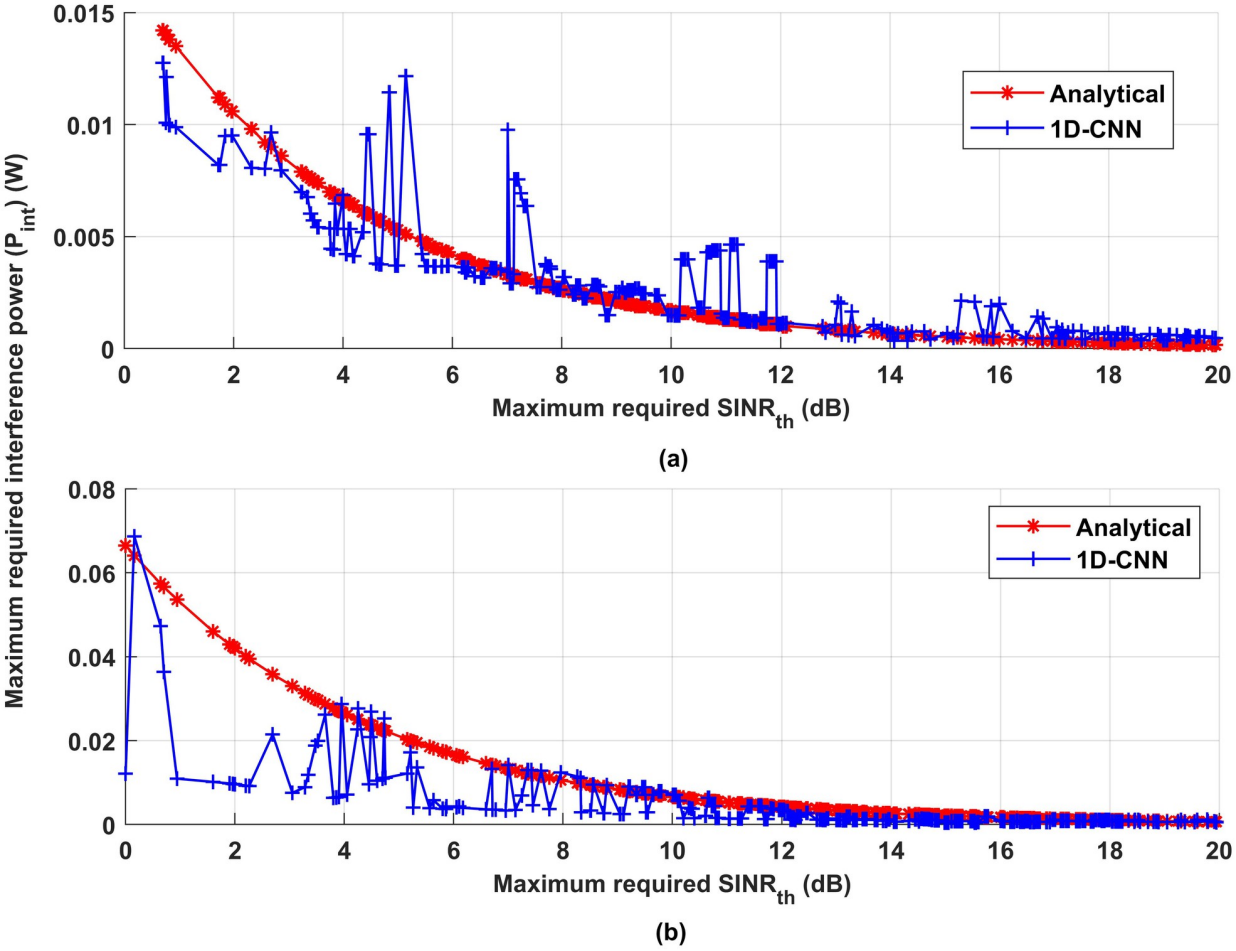
Maximum required *SINR*_*th*_ vs Maximum required interference power.

In order to attain the necessary system performance and obtain trustworthy data, whether at the driver or traffic management level, Fig 18 compares the required *SINR*_*th*_ vs the maximum required interference power (Pint) for the analytical and deep learning models. The distance between the IoT device and all of the receivers (*d*_*CX*_, *d*_*DX*_, and *d*_*VX*_)—including the driver and traffic management—is assumed to be identical to the distance between interfere devices and all of the receivers in order to assess the efficacy of the suggested model. As a result, Fig 18 shows that for both analytical and deep learning models, increasing the necessary *SINR*_*th*_ reduces interference transmission power. By lowering interference power, you may be lessening the influence of undesired signals in the context of IoT devices communication and facilitating the successful transmission of the intended signal. Reducing interference power may also help the receiver distinguish the desired signal from interference and background noise, allowing for faster and more effective communication between IoT devices and drivers or traffic management, which improves safety and smoother traffic flow.

**Fig 19:**
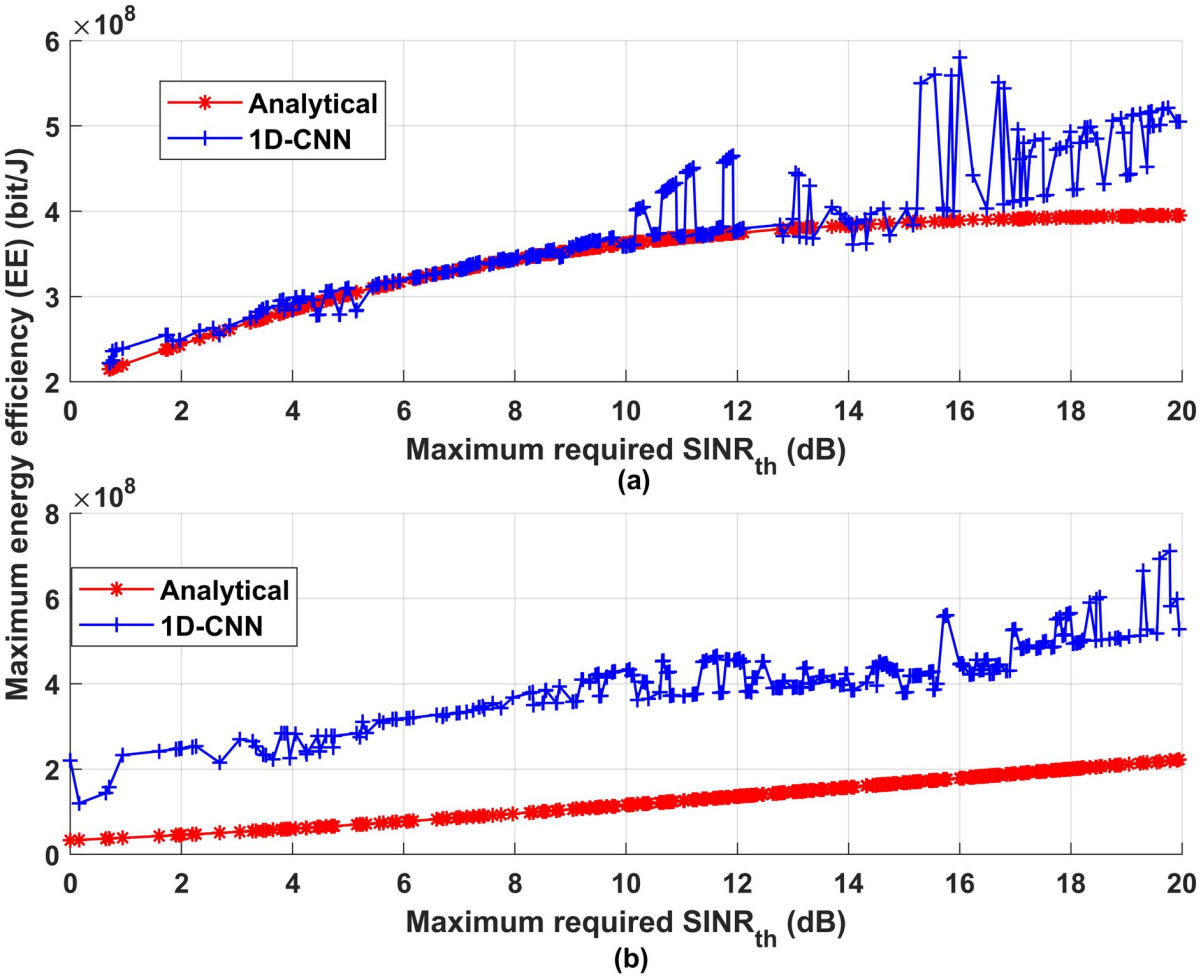
Maximum required *SINR*_*th*_ vs maximum energy efficiency (EE).

Another metric has been measured to show the effectiveness of the proposed communication model is the energy efficiency (EE). Fig 19 depicts the maximum required *SINR*_*th*_ vs the maximum energy efficiency. As can be noticed from Fig 19 increasing *SINR*_*th*_ increases maximum EE for both proposed analytical and deep learning model. In general, a greater *SINR*_*th*_ indicates that the received signal quality is superior to that of noise and interference. To reach a certain performance level, less transmit power is needed as *SINR*_*th*_ increases. The communication system uses less energy when signals are transmitted at lower power levels. In wireless communication systems where energy consumption is a crucial consideration, like in (IoT) devices or battery-powered devices, efficient transmission at lower power levels is especially important. In conclusion, the correlation between *SINR*_*th*_ and EE can be summed up as follows: higher *SINR*_*th*_ allow for better resource utilisation, lower transmission errors, and the application of energy-saving techniques, all of which enhance communication systems’ energy efficiency.

**Fig 20:**
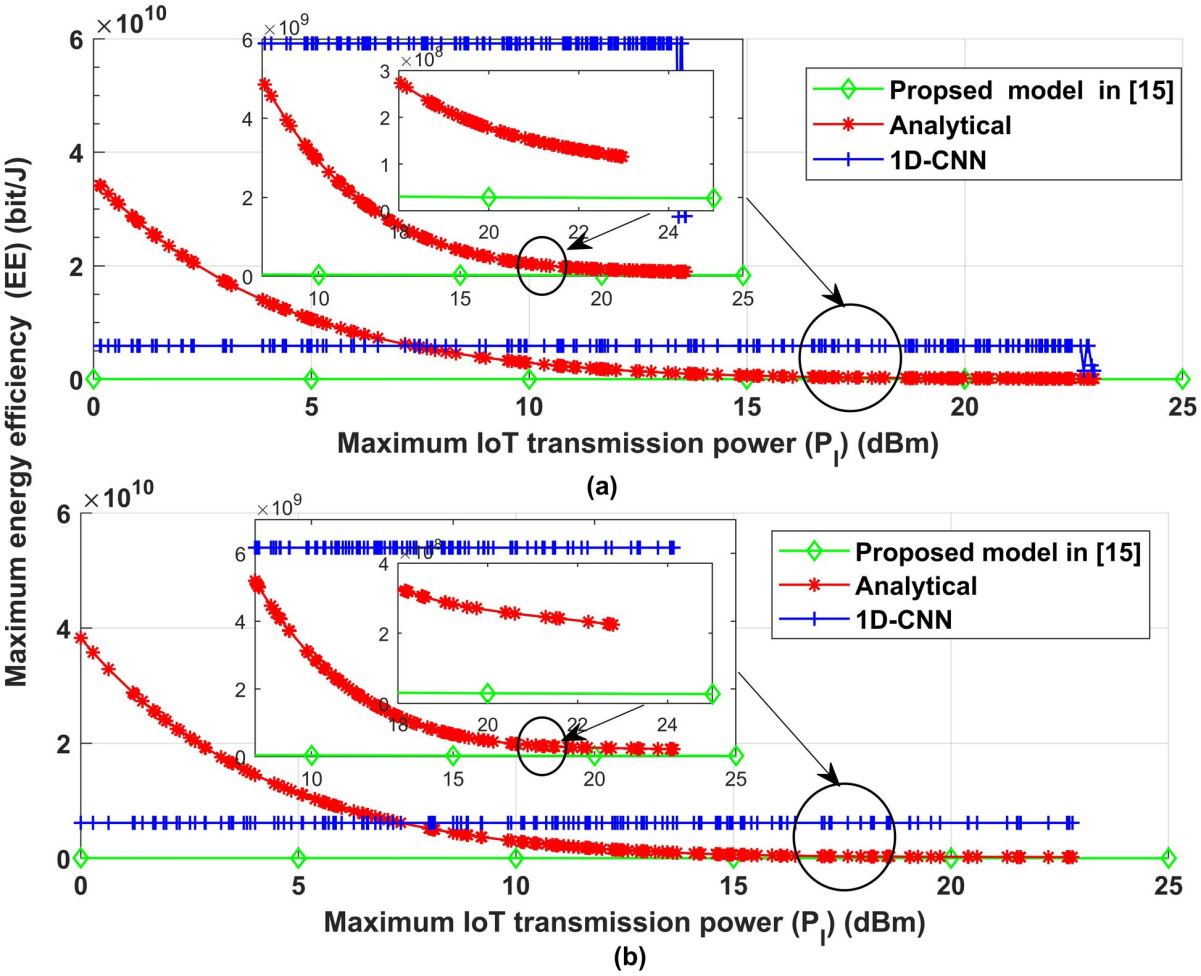
Maximum IoT transmission power vs maximum energy efficiency (EE).

To show the effectiveness of the proposed model, the proposed model has been compared with one of most recent published proposed [21]. Fig 20 illustrates the effect of IoT transmission power on the IoT system performance in terms of EE compared with the work proposed in [21]. Fig 20 shows the energy efficiency performance of our proposed model and the model proposed in [21] as a function of transmission power levels. Surprisingly, both the existing and current models reveal a similar trend: increasing gearbox power leads to a decrease in energy efficiency. Notably, across the entire range of transmission power levels, the proposed model consistently beats the model provided in [21]. This distinctive behavior showcases the effectiveness of the proposed technique in attaining higher energy efficiency results even when transmission power levels vary. The results illustrate the proposed suggested model’s creativity and increased performance, establishing it as a more energy-efficient alternative when compared to existing approaches.

From the proposed model and the results obtained it can be concluded that the efficiency of traffic flow can be greatly increased by integrating deep learning with IoT in traffic control. Traffic signals can be dynamically changed to optimise traffic flow, lessen congestion, and enhance overall road safety by utilising real-time data and predictive algorithms. Additionally, less fuel and pollutants might result from effective traffic management. This reduces the carbon footprint of urban mobility, which benefits the environment as well as the economy by saving money on fuel and maintenance. The suggested model may be adjusted to fit various city sizes and traffic situations, and it is flexible enough to be applied to other urban environments. Because of its modular design, implementation can be customized to meet local needs with flexibility. To further improve traffic management and urban mobility, the model can also be coupled with other smart city technologies, like enhanced sensor networks and driverless cars. In order to further enhance traffic management systems, future study may examine developments in deep learning approaches, such as more complex neural network designs or unique optimization algorithms. Further research and pilot programs will be necessary to evaluate the suggested system’s long-term dependability and efficacy in practical settings. System performance can be maintained and improved with ongoing monitoring and improvement based on real-world data.

## Conclusion

In conclusion, the suggested multimodal approach provides a thorough answer to Egypt’s urban traffic problems by fusing cutting-edge technology with better infrastructure. We seek to greatly increase traffic efficiency and road safety by putting a strong emphasis on improved driver-traffic system communication, real-time data collecting, and the deployment of cutting-edge technology such automated traffic violation systems. An advanced IoT communication network driven by distributed deep learning algorithms benefits the traffic management system, which includes detection cameras, Raspberry Pi 3 microcontrollers, PV modules, chargers, batteries, conventional traffic lights, an Android app, and cloud connectivity. This all-encompassing strategy aims to lessen traffic jams, shorten travel times, and bring Egypt’s urban mobility into compliance with international standards for intelligent transportation. We hope to make Egyptian communities’ futures more sustainable, effective, and livable by embracing these cutting-edge technology and improving traffic management techniques, which will ultimately result in a better, smarter urban environment.
